# Relationship Between Hemoglobin, Serum Iron, Iron-Binding Capacity, Ferritin, Zinc, Vitamin B12, and Vitamin D Levels in Patients with Melasma and the Severity of Their Condition

**DOI:** 10.5152/eurasianjmed.2026.251291

**Published:** 2026-04-10

**Authors:** Ebru Akal, Mehmet Melikoğlu

**Affiliations:** 1 Department of Dermatology and Venereology, Health Sciences University Erzurum Regional Training and Research Hospital, Erzurum, Türkiye; 2 Department of Dermatology, Atatürk University Faculty of Medicine, Erzurum, Türkiye

**Keywords:** Melasma, ferritin, vitamin D, vitamin B12, zinc

## Abstract

**Background:**

Melasma is an acquired disease with an unclear etiology. This study aimed to compare the hemoglobin, serum iron, iron-binding capacity, ferritin, zinc, vitamin B12, and vitamin D levels in patients with melasma to those in controls and to investigate a possible relationship between these parameters and melasma severity.

**Methods:**

One hundred consecutive patients with melasma and 50 healthy controls were included in this case–control observational study. Demographic features and disease duration were recorded. Melasma lesions were scored according to the melasma area severity index (MASI). Hemoglobin, serum iron, total iron binding capacity (TIBC), ferritin, vitamin D, vitamin B12, and zinc levels were compared between patients and controls. Possible correlations between these parameters and the MASI were also investigated.

**Results:**

In terms of laboratory data, there were statistically significant differences in hemoglobin, vitamin D, ferritin, and TIBC between melasma patients and the control group, but no statistically significant difference was observed in vitamin B12, zinc, and iron levels. A statistically significant positive correlation was found between the MASI score, age, melasma disease duration, and ferritin.

**Conclusion:**

The study presented lower hemoglobin, vitamin D, and ferritin levels in patients with melasma than in controls and demonstrated a relationship between lower ferritin levels and melasma severity. Considerations of these deficiencies may contribute to the holistic approach to melasma.

**Main Points**

- In this study, the authors aimed to shed light on treatment by examining the relationship between nutritional parameters and melasma, a disease whose etiology is not yet clearly understood.
- The study presented lower hemoglobin, vitamin D, and ferritin levels in patients with melasma than in controls and demonstrated a relationship between lower ferritin levels and melasma severity.
- The authors believe that correcting the nutritional parameter abnormalities detected in patients with melasma will contribute to the treatment of melasma.

## Introduction

Melasma is a common type of acquired hypermelanosis characterized by irregularly bordered and symmetrical patches of hyperpigmentation that appear only on sun-exposed areas. It most commonly appears on the face and less frequently on the neck and forearms. This condition is more common in dark-skinned women of African, Asian, and Hispanic descent.^1^ Although its exact prevalence is unknown due to racial and sun exposure differences, its estimated frequency is between 5% and 46%.^2^

Although many factors such as genetic predisposition, ultraviolet radiation exposure, and hormonal effects are implicated, its etiology has not been clearly elucidated.^3^ Conditions that increase the level of estrogen (pregnancy, oral contraceptives, hormone replacement therapies), cosmetics, photostimulant drugs, thyroid and liver dysfunctions, race, psychosomatic factors, and nutritional disorders can also cause melasma.^4^^-^^7^ Unclear etiopathogenesis causes difficulties in management, and treatment-related pigmentation disorders (hypohyperpigmentation) also pose an important problem.

Deficiencies in several vitamins (vitamin B12, vitamin D), Fe, and zinc have been well defined as factors related to some skin disorders.^8^^-^^12^ However, only a few studies have investigated these deficiencies in patients with melasma, indicating that appropriate replacement of these parameters might lead to more successful disease management.^8^^,^^13^^,^^14^ These parameters have essential roles in different physiological processes, such as cell differentiation, proliferation, apoptosis, purine and pyrimidine metabolism, and several enzymatic or transcriptional reactions. Their disturbances may lead to epidermal changes.^8^^,^^10^^,^^13^

In this study, the authors aimed to evaluate hemoglobin, iron, iron-binding capacity, ferritin, zinc, vitamin D, and vitamin B12 levels in patients with melasma and a control group and to determine a possible relationship between these parameters and the severity of melasma.

## Material and Methods

The research, approved by the ethics committee of Atatürk University on March 29, 2018, with decision number 3/43, was designed as a prospective case-control observational study conducted at the Dermatology Clinic of Atatürk University Faculty of Medicine between March 2018 and March 2019, in accordance with the principles of the Declaration of Helsinki. All patients were informed about the study, and their written consent was obtained. After calculating the sample size as 98 with 90% power and 95% confidence, 100 consecutive cases who were clinically diagnosed with melasma and age-gender matched 50 healthy controls (1/2 of the patients’ number) were included.^15^ Inclusion criteria for the patient group were clinical diagnosis of melasma, age between 18-60 years, acceptance of study conditions, and signing the informed consent form. The exclusion criteria included other diseases with facial hyperpigmentation, any malignant disease, and any drug or vitamin usage. Patients with any risk factors for melasma other than medication use were not considered within the exclusion criteria. The controls were individuals without known diseases and without any drug or vitamin usage among hospital staff.

Demographic features (age, sex), disease duration (months), and sunscreen usage were recorded.

Melasma lesions were evaluated clinically by standard lighting and scored according to the MASI. The MASI score is calculated by subjectively evaluating 3 factors in the face, right malar region, left malar region, and chin region. These factors are area of involvement, darkness, and homogeneity. For each of these 4 areas, a score between 0 and 6 is given for area of involvement and between 0 and 4 for darkness and homogeneity. For each of the 4 facial regions, the MASI score is obtained by summing the darkness and homogeneity values and multiplying them by the area of involvement value, and then adding them together. The total score ranges from 0 to 48.^15^

Blood samples from participants were analyzed for hemoglobin levels using a Sysmex XN-9000 device; iron, total iron-binding capacity (TIBC), ferritin, vitamin D, and vitamin B12 levels using a Beckman Coulter DXI 800 device; and zinc levels using a Beckman Coulter AU 5800 device. Considering laboratory reference values, the following lower limit values were accepted: 12 g/dL for hemoglobin in women and 14 g/dL in men; 200 pg/mL for vitamin B12; 11.5 µmol/mL (70 µg/dL) for zinc; 30 ng/mL for vitamin D; 30 ng/mL for ferritin; and 50 µg/dL for iron. Finally, a TIBC above 250 µg/dL was considered the upper limit.

Data, whose suitability for normal distribution was assessed using the Shapiro-Wilk test with means and standard deviations (SD), were statistically analyzed using SPSS for Windows 21.0 software (IBM Corp.; Armonk, NY, USA). Descriptive frequencies were calculated for demographic and clinical variables. Categorical variables are presented as numbers and percentages. Student’s *t-*tests, Mann–Whitney *U*-tests, Kruskal–Wallis tests, and chi-squared tests were performed to compare the mean values between the groups and to compare categorical variables. Correlation analysis was performed by the Spearman test to determine the rho coefficient and level of significance. *P *< .05 was considered statistically significant.

## Results

There were 95 female (95%) patients, 5 male patients with melasma and 47 female-3 male controls. The mean age was 31.9 ± 8.5 years (mean ± SD) in patients and 30 ± 9.4 years (mean ± SD) in controls (*P *> .05). The disease duration was 1.4 ± 0.6 years. Sunscreen usage was present in 32 cases with melasma (32%) and 8 controls (16%) (*P *= .050).

In terms of laboratory data, there were statistically significant differences in hemoglobin, hematocrit, RBC, MCH, MCHC, PLT, vitamin D, ferritin, and TIBC between cases and controls. In the patient group, vitamin B12, zinc, and iron levels were found to be lower compared to the control group, but the difference was not statistically significant. The data are shown in Table 1.

The mean MASI score was 11.1 ± 5.8 (mean ± SD) in patients. A statistically significant positive correlation was found between the MASI score, age, melasma disease duration, and ferritin. The correlation analysis is shown in Table 2.

## Discussion

Melasma is a common dermatological disease whose etiology has not yet been clarified. Although the literature includes studies on some accompanying diseases and triggering factors, there are very few studies on the necessity of laboratory tests that can shed light on melasma treatment options.

Evaluating CBC parameters of the patients and controls in this study, there were statistically significant differences in hemoglobin, hematocrit, MCH, MCHC, RBC, and platelet count between patients and controls. Additionally, there were statistically significant differences in serum ferritin levels and TIBC between groups, indicating iron deficiency anemia in patients. Although iron deficiency and iron deficiency anemia have been investigated in many dermatological diseases, few studies have examined the relationship between iron deficiency and melasma.^16^ Najad et al’s^16^ study investigating the prevalence of anemia in patients with melasma and Behrangi et al’s^17^ study investigating iron and ferritin levels in melasma patients have revealed a clear link between melasma and iron deficiency. Although a clear link between iron deficiency and the development of melasma has not been demonstrated, several mechanisms have been implicated. Since iron is a cofactor for tyrosinase, the main enzyme in melanogenesis, it is thought that iron deficiency may lead to changes in melanin synthesis and hyperpigmentation. Additionally, oxidative stress caused by reactive oxygen species that arise in iron deficiency can increase melanocyte activity, leading to hyperpigmentation.^18^ Furthermore, Qazi et al^19^ found lower hemoglobin and ferritin levels in melasma patients compared to the control group and also demonstrated a negative correlation between MASI and these parameters. Based on the results of their study, they suggested that the serum iron profile has an important role in the etiopathogenesis of melasma and that adding iron replacement to its management will increase the clinical outcome. Similarly, the negative correlation that was demonstrated between serum ferritin levels and the MASI score was statistically significant. The hypothesis of a strong relationship between melasma and anemia was confirmed by the fact that ferritin and iron values were lower and TIBC values were higher in the patient group and by the negative correlation between MASI scores and ferritin levels in this study. Although not statistically significant, the lower average iron levels in the patient group compared to the control group may indicate that the relationship between melasma and iron deficiency should be investigated in studies with more participants.

There are very few studies evaluating the link between melasma and vitamin D levels. Chaitanya et al^13^ investigated the frequency of vitamin D abnormalities, periodontitis, and anemia in melasma patients. A significant relationship has been found between melasma, vitamin D levels, and periodontitis. However, this relationship was not observed for hemoglobin values, and they suggested that this association might be syndromic. A different study suggested that vitamin D deficiency, which may occur with sun avoidance and sunscreen creams, can be overcome with oral vitamin D supplementation since exposure to sunlight may increase melasma lesions.^20^ Furthermore, since the skin is the primary site for vitamin D production and the vitamin D receptor is found in both epithelial cells and melanocytes, it has been reported that when 1,25(OH)2D3 binds to melanocytes, it may cause a decrease in melanocyte proliferation.^13^ This study, similar to other studies, found that melasma patients had low vitamin D levels and high sunscreen use. The authors believe that vitamin D deficiency may play a role in the etiopathogenesis of melasma and that melasma patients are more sensitive to sunscreen use.

Although the study found lower B12 levels in patients with melasma, this difference was not considered statistically significant. In vitamin B12 deficiency, which is a cofactor for enzymatic reactions, atrophic glossitis, recurrent aphthous stomatitis, and hair and nail changes can be observed, but skin findings are not frequently seen. However, prominent hyperpigmentation in the flexural areas, palms, soles, nails, oral mucosa, and rarely diffuse or melasma-like hyperpigmentation can be seen.^8^^,^^21^ Case-based studies presenting with facial macular pigmentation, melanonychia, and bilateral inguinal and palmar hyperpigmentation, poikiloderma-like hyperpigmentation, and tongue pigmentation have identified B12 deficiency and observed regression of these findings with B12 replacement alone. Studies linking the mechanism of hyperpigmentation formation in B12 deficiency to low glutathione levels and increased phenylalanine levels in patients with B12 deficiency exist. Glutathione inhibits melanogenesis by inhibiting the effect of tyrosinase; therefore, melanogenesis cannot be inhibited at low glutathione levels. Furthermore, it is known that increased phenylalanine increases melanin synthesis.^22^ ^-^^26^When the authors evaluated patients according to their vitamin B12 levels (below and above 200 mg/dL), 43% of the patients and 30% of the controls had vitamin B12 levels below 200 mg/dL, and 57% of patients and 70% of controls had vitamin B12 levels above 200 mg/dL. The detection of B12 deficiency in 43% of the patients is an important finding suggesting that B12 deficiency should not be ignored in its relationship with melasma. The authors believe that more comprehensive studies will more clearly reveal the relationship between melasma and B12 deficiency, and furthermore, the benefits of B12 replacement in the treatment of melasma.

Zinc is both a cofactor in enzymatic reactions and essential for many transcription factors. In a study by Sharquie et al^27^ and Kirsch et al,^28^ in which they evaluated the effectiveness of 10% zinc oxide-containing topical creams on melasma, it was suggested that topical zinc oxide was influential in the treatment. In contrast, in studies by Yousefi et al^29^ and Iraji et al,^30^ in which they compared topical 4% hydroquinone and 10% zinc sulfate for melasma treatment, it was reported that the use of topical zinc sulfate alone is insufficient in the treatment of melasma. In their cross-sectional study (including 118 melasma patients and 118 control subjects) examining the relationship between serum zinc levels and melasma, Mogaddam et al^14^ reported that serum zinc levels were significantly higher in the control group. In this study, zinc deficiency was detected in 67% of patients, and patients with low zinc levels had higher average MASI scores. Although not statistically significant, this difference suggests that further studies with larger sample groups may be needed regarding the relationship between zinc and melasma.

This study demonstrated lower hemoglobin, ferritin, and vitamin D levels in melasma patients than in controls. The significant correlation between low ferritin levels and melasma severity may indicate a link between these parameters and melasma. Although some studies suggest replacing vitamin D or iron in melasma cases with these deficiencies, further studies specifically designed to evaluate the effects of these replacements in melasma itself will be needed to address them as therapeutic targets for melasma.^19^^,^^20^

The study results may have both limitations and strengths in some aspects. The main limitation of this study is that the melasma cohort was from a single tertiary care center and therefore does not represent the entire spectrum. Another limitation of the study may be that seasonal effects were not taken into account when evaluating vitamin D levels. However, evaluating the relationship between these parameters and MASI, and identifying a significant correlation between low serum ferritin levels and melasma severity for the first time, could be one of the strengths of this study.

In conclusion, this study presented lower hemoglobin, vitamin D, and ferritin levels in patients with melasma than in controls and demonstrated a relationship between lower ferritin levels and melasma severity. Considerations of these deficiencies may contribute to the holistic approach to melasma.

### Artificial Intelligence Usage Statement:

The authors declared that no Artificial Intelligence Tool was used in the preparation of the manuscript.

## Figures and Tables

**Table 1.** Comparison of Laboratory Parameters of Cases and Controls

**Characteristics**	**Cases (** **100** **)**	**Controls (** **50** **)**	***P***
Gender			NS
Female	95	47	
Male	5	3	
Age			NS
Years (mean ± SD)	31.9 ± 8.5	30 ± 9.4	
WBC^1^	8064.1 ± 1790.8	8268.2 ± 3286.6	.680^a^
RBC^1^	4929 ± 424	5193 ± 615	.008^b^
HGB^1^	13.4 ± 1.4	14.5 ± 1.7	<.001^b^
HGB n (%)			.664^c^
Low	14(%14)	5 (%10)	
Normal	86 (%86)	45 (%90)	
HCT^2^	41.4 ± 3.9	43.2 ± 4.4	.011^b^
MCV^1^	84.4 ± 5.7	82.7 ± 10.6	.812^a^
MCH**^1^**	27.2 ± 2.5	28.1 ± 2.9	.005^a^
MCHC**^1^**	32 ± 1.3	34.3 ± 7.5	<.001^a^
PLT**^1^**	303 790 ± 62 756	278 220 ± 61 772	.022^a^
Zinc^1^	10.5 ± 3.8	11.4 ± 3.2	.160^b^
Zinc n (%)			.108^c^
11.5 and below	67 (%67)	26 (%52)	
Over 11.5	33(%33)	24 (%48)	
Vitamin B12^1^	244.9 ± 140.8	263.2 ± 114.3	.131^a^
Vitamin B12, n (%)			.173^c^
200 and below	43 (%43)	15 (%30)	
Over 200	57 (%57)	35 (%70)	
Vitamin D^1^	13.2 ± 10.3	15.5 ± 8.3	.017^a^
Vitamin D, n (%)			
30 and below	95 (%95)	48 (%96)	1.00^c^
Over 30	5 (%5)	2 (%4)	
Ferritin^1^	24 ± 28.4	60.7 ± 60.1	<.001^a^
Ferritin, n (%)			.001**^c^**
30 and below	76 (%76)	23 (%46)	
Over 30	24 (%24)	27 (%54)	
Iron^1^	59.5 ± 35.2	71.5 ± 38.9	.059^b^
Iron, n (%)			.114^c^
50 and below	47 (%47)	16 (%32)	
Over 50	53 (%53)	34 (%68)	
TIBC^1^	318.3 ± 76.2	270.1 ± 66.3	<.001^b^
TIBC			<.001^c^
250 and below	16 (%16)	22 (%44)	
Over 250	84 (%84)	28 (%28)	

^1^ Mean ± standard deviation.

^a^ Mann–Whitney *U*-test; ^b^ Independent Sample *T* Test; ^c^ Yates Continuity Correction.

WBC, white blood cell; RBC, red blood cell; HGB, hemoglobin; HCT, hematocrit; MCV, mean corpuscular volume; MCH, mean corpuscular hemoglobin; MCHC, mean corpuscular hemoglobin concentration; PLT, platelet; TIBC, total iron-binding capacity.

**Table 2.** Relationship Between the MASI and Continuous Data

**Characteristics**	MASI* ** r**	***P***
Age	0.233**	<.05
Duration of melasma	0.203**	<.05
WBC	0.045	NS
RBC	0.157	NS
HGB	0.174	NS
HCT	0.161	NS
MCV	0.026	NS
MCH	0.040	NS
MCHC	0.040	NS
PLT	−0.107	NS
Zinc	−0.054	NS
Vitamin B12	0.078	NS
Vitamin D	0.121	NS
Ferritin	−0.287^**^	<.05
Iron	−0.062	NS
TIBC	−0.069	NS

*Spearman Correlation **P < .05.

MASI, melasma area severity index.

## Data Availability

The data that support the findings of this study are available on request from the corresponding author.
